# The effects of starter microbiota and the early life feeding of medium chain triglycerides on the gastric transcriptome profile of 2- or 3-week-old cesarean delivered piglets

**DOI:** 10.1186/s40104-017-0213-1

**Published:** 2017-11-02

**Authors:** Paolo Trevisi, Davide Priori, Vincenzo Motta, Diana Luise, Alfons J. M. Jansman, Sietse-Jan Koopmans, Paolo Bosi

**Affiliations:** 1 DISTAL, University of Bologna, Viale Fanin 46, 40127 Bologna, Italy; 2 Wageningen University and Research, Wageningen, The Netherlands

**Keywords:** Development, Microbiota, Pig, Stomach, Transcriptome

## Abstract

**Background:**

The stomach is an underestimated key interface between the ingesta and the digestive system, affecting the digestion and playing an important role in several endocrine functions. The quality of starter microbiota and the early life feeding of medium chain triglycerides may affect porcine gastric maturation. Two trials (T1, T2) were carried out on 12 and 24 cesarean-delivered piglets (birth, d0), divided over two microbiota treatments, but slaughtered and sampled at two or three weeks of age, respectively. All piglets were fed orally: sow serum (T1) or pasteurized sow colostrum (T2) on d0; simple starter microbiota (*Lactobacillus amylovorus*, *Clostridium glycolicum* and *Parabacteroides* spp.) (d1-d3); complex microbiota inoculum (sow diluted feces, CA) or a placebo (simple association, SA) (d3-d4) and milk replacer ad libitum (d0-d4). The The T1 piglets and half of the T2 piglets were then fed a moist diet (CTRL); the remaining half of the T2 piglets were fed the CTRL diet fortified with medium chain triglycerides and 7% coconut oil (MCT). Total mRNA from the oxyntic mucosa was analyzed using Affymetrix©Porcine Gene array strips. Exploratory functional analysis of the resulting values was carried out using Gene Set Enrichment Analysis.

**Results:**

Complex microbiota upregulated 11 gene sets in piglets of each age group vs. SA. Of these sets, 6 were upregulated at both ages, including the set of gene markers of oxyntic mucosa. In comparison with the piglets receiving SA, the CA enriched the genes in the sets related to interferon response when the CTRL diet was given while the same sets were impoverished by CA with the MCT diet.

**Conclusions:**

Early colonization with a complex starter microbiota promoted the functional maturation of the oxyntic mucosa in an age-dependent manner. The dietary fatty acid source may have affected the recruitment and the maturation of the immune cells, particularly when the piglets were early associated with a simplified starter microbiota.

## Background

The stomach is a key point in the interface between the ingesta and the digestive system, affecting post-gastric efficiency of digestion, playing a role in several endocrine functions and being involved in several diseases of domestic animals and humans. Nevertheless the main molecular-cellular processes that govern its development and homeostasis have not yet been well defined. Current evidence indicates that the stomach can actively interact with the microbiota resident in or in transit to the lower digestive tracts. In pigs, the stomach shows an array of important tools related to immunity and barrier defense, also developmentally controlled: toll-like receptors (TLRs) 2, 3 and 4 [1] among the receptors specialized in detecting microorganisms and activating an innate immune response, the polymeric immunoglobulin receptor responsible for the transport of secretory immunoglobulins [1], submucosal and mucosal lymphoid follicles with and without compartmental organization of T and B lymphocytes [2] and plasma cells [3]. This implies the relevance of adequate stimulation of the local microbiota as has been demonstrated in the intestine [4]. This would also have practical relevance for managing the piglets, namely stimulating the diffusion of management solutions favoring the acquisition by neonate piglets of microbiota also out of their litter. Conversely, the acquisition of the dominant importance of specific core microbiota would indicate that certain bacterial pools can be used for the early orientation or acceleration of gut maturation. However, surprisingly, studies regarding the impact of different microbiota on the development of the stomach of mammals are lacking. It has generally been recognized that the availability of a single ‘standard’ microbiome could circumvent several problems in studies focusing on the interaction of the host and its microbiota [5]. For this purpose, Laycock et al. [6] selected a few bacteria strains as a standardized microbiota owing to their positive influence on a new early microbial colonization in the porcine gut.

Medium chain triglycerides have received research attention due to their potential ability for selecting microbes [7, 8]; they are an interesting nutrient source for very young animals [9], but also potentially interact with the acid-secreting function of the oxyntic mucosa, owing to their acidic nature. It has been observed that constant dietary supplementation with different dietary organic acids can affect (Formate [10]) or not (Butyrate [11]) the morphology of the oxyntic mucosa of the young pig, reducing the relative number of parietal cells responsible for acid secretion, and the expression of the protein marker of this function, H^+^/K^+^-ATPase. This could be due to the reduced gastrin-dependent growth stimulation of the oxyntic cells after the increase in feed acidity [12].

Two studies were designed to elucidate the molecular networks in the oxyntic mucosa of the young pig which characterize the long-term individual response after various associations with a simplified or complex starter microbiota during their first days of life. Furthermore, an additional aim in the second study was to evidence the possible action of a dietary source of medium chain triglycerides on the transcriptome of the oxyntic mucosa.

## Methods

**Fig. 1:**
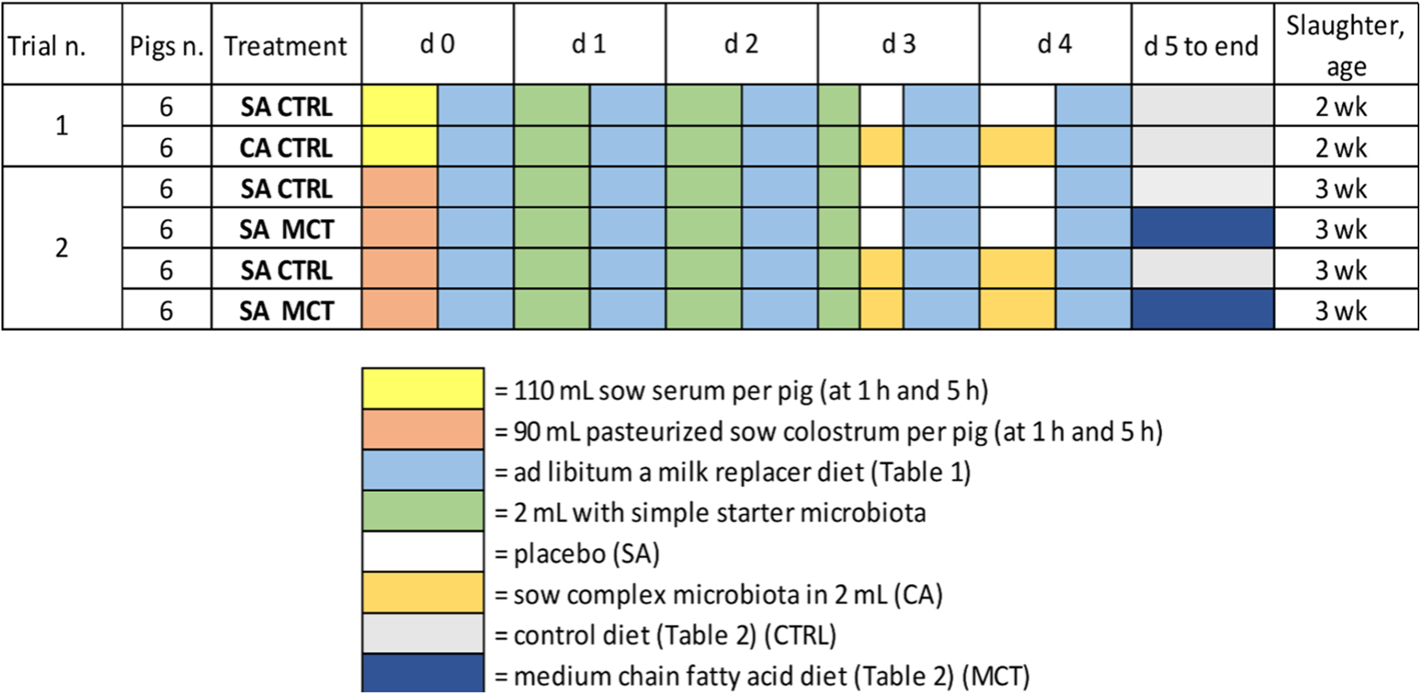
General summary of the factors in the two trials

Two different trials (Fig. 1) were carried out on 12 and 24 piglets obtained from from 4 and 6 sows respectively [(Great York × Pietrain) × ‘Dalland’ cross] by cesarean delivery (CD) (d 0); they were divided over two microbiota association treatments, and were then slaughtered and sampled at wk 2 (Trial 1) or wk 3 (Trial 2) of age, respectively. Piglets from different litters were balanced over the various groups based on litter, body weight and gender and in each pen/room 3 piglets per group were housed.

The piglets were housed in two pens per clean, non-sterile room having an automatic feeding system for supplying a moist diet, and were balanced for birth weight (BW) and litter of origin. At 1 h and 5 h after birth, each piglet received 110 mL sow serum (Trial 1) or 90 mL pasteurized (30 min at 60 °C) sow colostrum by oral gavage [13, 14] (Trial 2). All the piglets received a simple starter microbiota consisting of *Lactobacillus amylovorus* (3.6 × 10^7^ CFU), *Clostridium glycolicum* (5.7 × 10^7^ CFU) and *Parabacteroides* spp. (4.8 × 10^7^ CFU) by oral inoculation (2 mL) on d1, d2 and d3 after birth. These bacteria are the most frequently identified of the phylogenetic groups in the intestines of piglets as have previously been proposed as minimal intestinal colonization microbiota for gnotobiotic pigs [6].

On d 3 (at 15:30 and 21:00 h) and on d 4 (at 8:00 h), the piglets of treatment CA received a fecal-oral inoculant immediately after feeding in the form of a 2-mL suspension of feces (feces in 0.1 mol/L sterile potassium phosphate buffer (pH 7.2), diluted 1:10). The SA-treated piglets received a placebo treatment (2 mL of a sterile 0.1 mol/L potassium phosphate buffer, pH 7.2).

Average birth weight was 1213 ± 144 g and 1228 ± 164 (Trial 1), and 1273 ± 138 g and 1275 ± 153 g (Trial 2) for the CA and the SA piglets, respectively. The piglets were fed a milk replacer diet ad libitum during a period of 5 d (d0 - d4). It consisted of bovine skimmed milk powder, whey powder, vegetable oil, hydrolyzed wheat protein, wheat starch, sucrose, and a vitamin and mineral premix, and contained 230 g crude protein per kg milk replacer (Table 1). All the piglets in Trial 1 and half of the piglets in Trial 2 were then fed a moist diet (CTRL) based on whey powder, maize, wheat, toasted full fat soybeans, oat flakes, sucrose, soybean meal, vegetable oil, coconut oil, wheat gluten, potato protein, rice protein, and brewer’s yeast during the remainder of the study (Table 2). The other half of the piglets in Trial 2 were fed the same diet but containing 7% of coconut oil in place of 4.68% soy oil and of 2.32% palm oil in order to generate a diet fortified in medium chain triglycerides (MCTs) (Table 2). In other words, the 24 piglets of the 2^nd^ trial were allocated by BW to 4 treatments arranged in a 2 × 2 factorial design, with 2 different early associations with microbiota (CA and SA) and two diets (CTRL and MCT). On d 16 (Trial 1), and on d 21 and d 22 (Trial 2), the piglets were euthanized, and the stomach was sampled always in the center of the area corresponding to the oxyntic mucosa.

**Table 1:** Analyzed nutrient composition of the artificial milk provided to the piglets during the first 4-day after birth (in g/kg, unless stated otherwise)

Nutrient	Content
Dry matter	957
Ash	78
Crude protein	222
Crude fiber	1
Starch	27
Glucose	29
Sugar	435
Lactose	405
Phosphorus	7.2
Calcium	8.6
Crude fat	227
Total fatty acids (g/kg fat)	913
Saturated fatty acids (g/kg fat)	461
Monounsaturated fatty acids (g/kg fat)	360
Polyunsaturated fatty acids (g/kg fat)	86

**Table 2:** Ingredient composition of the experimental diets provided to the piglets from day 5 after birth until the end of the study (g/kg)

Ingredients	CTRL	MCT
Maize	200.0	200.0
Whey powder	180.0	180.0
Wheat	150.0	150.0
Oat hulls	80.0	80.0
Barley	70.0	70.0
Wheat gluten meal	50.0	50.0
Soybeans, heat treated	40.0	40.0
Soycomill-P	33.0	33.0
Lactose	30.0	30.0
Protastar	30.0	30.0
Monocalciumphosphate	0.6	0.6
Rice meal	12.0	12.0
Limestone	0.9	0.9
Salt	3.6	3.6
Dicalciumphosphate	19.4	19.4
Sodium bicarbonate	4.5	4.5
Piglet premix 0–5%	5.0	5.0
*L*-Lysine HCl	5.9	5.9
*DL*-Methionine	2.0	2.0
*L*-Threonine	1.1	1.1
*L*-Tryptophan	0.9	0.9
*L*-Valine	0.5	0.5
Soy oil	50.6	3.8
Palm oil	30.0	6.8
Coconut oil	0	70.0

### RNA isolation and gene array quantification

Total RNA was isolated from the gastric tissue samples using the FastPure RNA kit (TaKaRa Bio Inc., Shiga, Japan). All other procedures were in agreement with the manufacturer’s protocol. The RNA purity and integrity were evaluated using Agilent Bioanalyzer 2100 (Agilent Technologies, Palo Alto, CA) just before the transcriptome analysis. The total RNA was hybridized on Affymetrix Porcine Gene 1.1 ST array strips. The hybridized arrays were scanned on a GeneAtlas imaging station (Affymetrix, Santa Clara, CA, USA). Performance quality tests of the arrays including the labelling, hybridization, scanning and background signals using Robust Multichip Analysis were carried out on the CEL files using the Affymetrix Expression Console.

### Statistics

The Affymetrix Trascript IDs, in general each characterized by several exonic sequences, were associated with 13,406 human gene names, based on *Sus scrofa* Ensembl (release 79, www.ensembl.org), where data regarding each porcine gene could be directly accessed using the names of genes which were adopted for the present text. Regarding the processed gene expression values, two exploratory functional analyses were applied with Gene Set Enrichment Analysis using the Hallmark (aggregating many gene sets to represent well-defined biological states or processes) and C5.BP (based on Gene Ontology (GO)) catalogues of gene sets from Molecular Signatures Database v5.1 (http://software.broadinstitute.org/gsea/msigdb/). The normalized enrichment score (NES) was calculated for each gene set, and statistical significance was defined when a False Discovery Rate (FDR) % < 25 and *P*-values of NES < 0.05. Enrichment score *P*- values were estimated using a gene set-based permutation test procedure. A new data set was created and added to the Hallmark catalogue which included 19 genes typically characterizing the stomach as compared with the different intestinal sections [15], and were more or equally expressed in the porcine oxyntic mucosa as compared with pyloric mucosa [16]. Reference sequences for all these gene are available on *Sus scrofa* Ensembl except for the following genes that have the Ensembl gene accession number: *ATP4A*, ENSSSCT00000003207; *EGF*, ENSSSCT00000010002, or the NCBI Reference Sequence: *LPL,* NM_214286.1; *TFF1*, XM_003358973.3.

The overlap in enriched GO terms was visualized using the Enrichment Map (http://baderlab.org/Software/EnrichmentMap20) plugin for Cytoscape 2.8.0 (http://www.cytoscape.org/), including the gene sets with a *P*-value <0.005 and an FDR *q*-value <0.10. The nodes were joined if the overlap coefficient was ≥0.5.

## Results

As general information, body weight gain was similar among piglet groups both in trial 1 and trial 2. Average birth weight was approx. 1 kg, increased to 2.5 kg at 2 wk of age and to 4 kg at 3 wk of age. Feed intake on a pen base did not differ. The general health score (including diarrhea) was similar among piglets, both for trial 1 and 2, and diarrhea was absent in trial 1 and in trial 2 diarrhea was only incidental and erratic among the 4 groups of piglets as judged on a daily basis from birth till 3 wk of age.

**Fig. 2:**
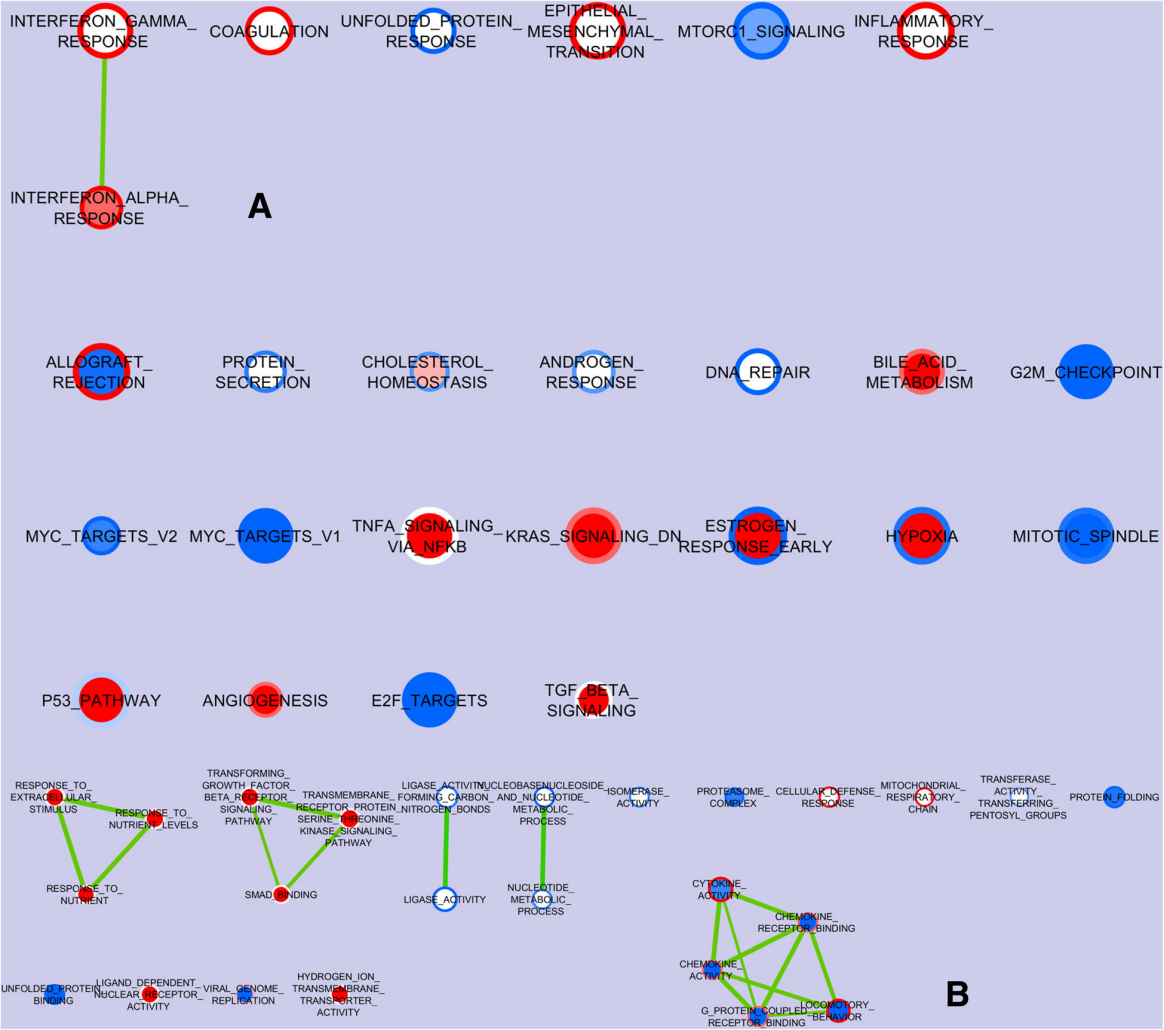
The nodes of the gene sets enriched in the oxyntic mucosa of piglets early associated with CA as compared to the SA piglets and sampled at two or three weeks of age. **a** The entire picture regarding the Hallmark gene set, summarizing the main biological states or processes; **b** Picture of nodes obtained with the more detailed gene ontology (GO) gene set after the removal of those related to cell mitosis; the nodes represent gene sets. The color on the center of the node represents the results of the trial ending at 2 wk; the color on the ring visualizes the trial ending at 3 wk. The edges represent the link of two or more gene sets sharing the same core group of genes explaining the enrichment of each of the gene sets. Enrichment significance (*P*-value) is conveyed as node color intensity where red stands for upregulation at 2 or 3 wk in the CA piglets, and blue stands for downregulation as compared with the SA piglets. The node size represents the number of genes in the gene set

Figure 2a visualizes the nodes of the gene sets of the Hallmark catalogue, enriched (color red) or impoverished (color blue) in the porcine oxyntic mucosa of piglets early associated with complex microbiota (CA) as compared to simplex microbiota (SA) piglets, and sampled at two or three weeks of age (represented by the center and the ring of each node, respectively). The CA upregulated 9 and 10 gene sets in piglets of two and three weeks of age, respectively as compared to the SA. Of these sets, 4 were upregulated at both ages. The CA downregulated 6 gene sets at both ages, all related to cycling activity, mitosis regulation steps and general protein synthesis. In addition, another 1 and 8 gene sets were downregulated in piglets of two and three weeks of age, respectively as compared to the SA.

In addition to the gene sets of the Hallmark catalogue, represented in Fig. 2a, the new cured gene set oxyntic mucosa was upregulated by the CA at both two and three weeks of age (Table 3). Nevertheless, the upregulation involved more genes in two-week old piglets than in the older piglets. Potassium voltage-gated channel, Isk-related family, member 2 (*KCNE2*), epidermal growth factor (*EGF*), Anion exchanger 2 (*SLC4A2*), Lipoprotein lipase (*LPL*), potassium inwardly rectifying channel, subfamily J, member 15 (*KCNJ15*) and solute carrier family 2 (facilitated glucose transporter), member 4 (*SLC2A4*) genes were enriched at both ages of the piglets.

**Table 3:** List of genes enriched or not inside the new cured gene set oxyntic mucosa tested inside the Hallmark catalogue

		Trial 1 (2-week-old piglets)		Trial 2 (3-week-old piglets)	
Gene product	Gene name	Rank enrichment score^a^	Core enrichment	Rank enrichment score^a^	Core enrichment
Potassium voltage-gated channel, Isk-related family, member 2	*KCNE2*	0.729	Yes	0.330	Yes
Epidermal growth factor	*EGF*	0.583	Yes	0.185	Yes
Anion exchanger 2	*SLC4A2*	0.580	Yes	0.176	Yes
Lipoprotein lipase	*LPL*	0.578	Yes	0.321	Yes
Potassium inwardly rectifying channel, subfamily J, member 15	*KCNJ15*	0.576	Yes	0.406	Yes
Chloride intracellular channel 6	*CLIC6*	0.495	Yes	0.070	No
Solute carrier family 2 (facilitated glucose transporter), member 4	*SLC2A4*	0.479	Yes	0.275	Yes
ATPase, H^+^/K^+^ exchanging, β polypeptide	*ATP4B*	0.417	Yes	0.058	No
ATPase, H^+^/K^+^ exchanging, α polypeptide	*ATP4A*	0.371	Yes	0.013	No
Insulin-like growth factor binding protein 5	*IGFBP5*	0.266	Yes	0.055	No
Pheromaxein, subunit C	*PHEROC*	0.248	Yes	−0.048	No
Acquaporin 4	*AQP4*	0.236	Yes	0.006	No
Potassium inwardly rectifying channel, subfamily J, member 13	*KCNJ13*	0.167	No	0.202	Yes
Ghrelin	*GHRL*	0.094	No	−0.237	No
Trefoil factor 1	*TFF1*	0.093	No	0.002	No
Pepsinogen C	*PGC*	0.075	No	−0.072	No
Insulin-like growth factor 1	*IGF1*	0.067	No	0.401	Yes
Pepsinogen B	*PGB*	−0.023	No	−0.202	No
Chitinase, acidic	*CHIA*	−0.089	No	−0.121	No

^a^Reflects the relative degree to which a gene is overrepresented at the top (positive sign) or bottom (negative sign) of the ordered dataset

Figure 2b visualizes the nodes of the gene sets of the C5 catalogue (based on Gene Ontology), differentially enriched in the CA or the SA piglets at 2 or 3 wk of age following the same scheme as Fig. 2a. From the figure, all the gene sets related to cycling activity and mitosis regulation, already shown in Fig. 2a, were depicted. The figure primarily shows that two groups of gene sets, related respectively to response to nutrient stimulus and to transforming the growth factor β (TGFB) pathway were upregulated by the CA in 2-week-old piglets, but not in older pigs; a group of gene sets related to chemokine activity was down- and upregulated with CA in piglets of two and three weeks of age respectively as compared to the SA.

**Fig. 3:**
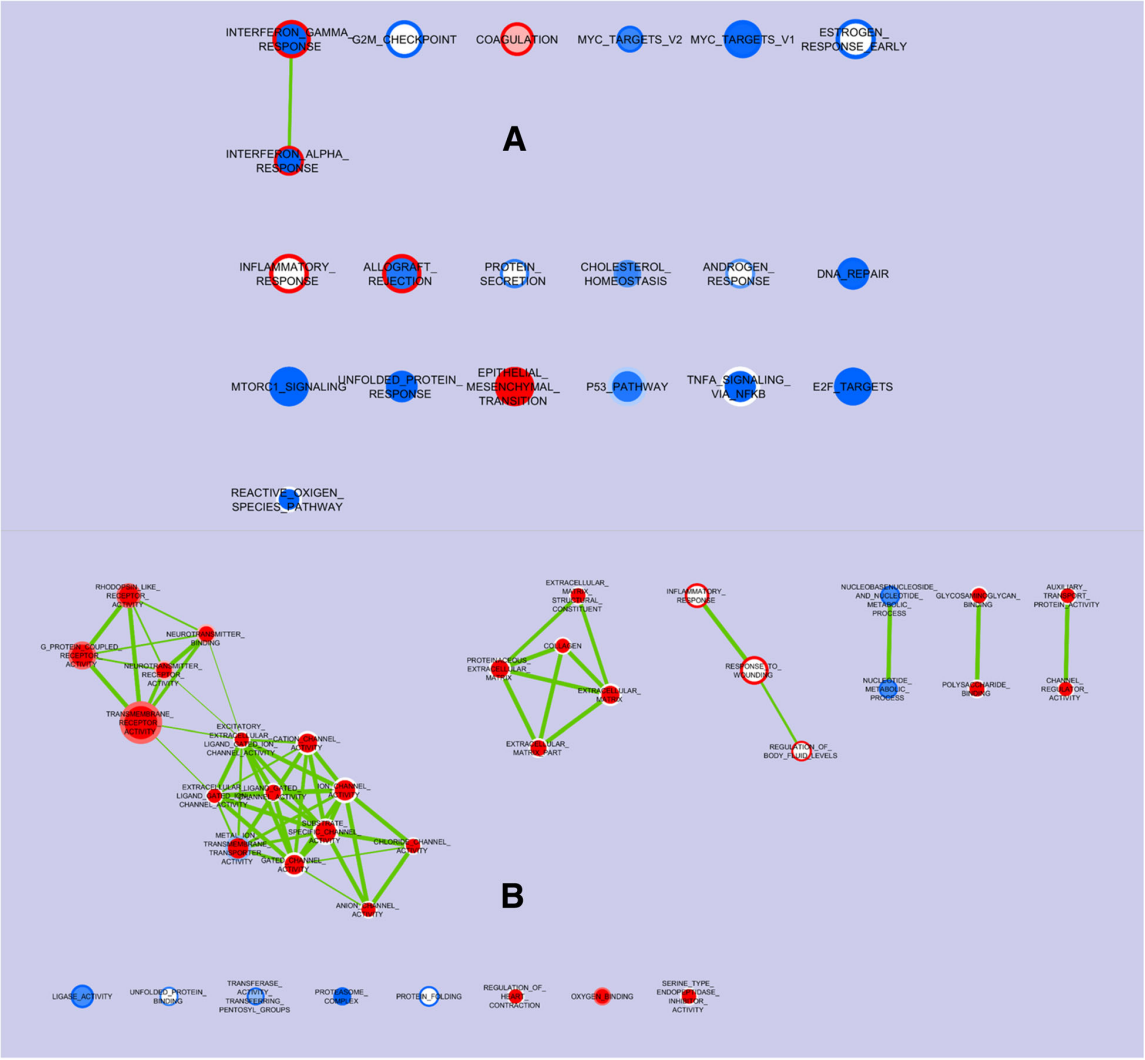
The nodes of the gene sets enriched in the oxyntic mucosa of piglets early associated with the CA as compared to the SA piglets, and supplemented with medium chain fatty acids (MCTs) or not (CTRL). **a** The entire picture for the Hallmark gene set, summarizing the main biological states or processes; **b** Picture of nodes obtained with the more detailed GO gene set; Nodes represent gene sets. The color on the center of the node represents the MCT diet; the color on the ring represents the CTRL diet. The edges represent the link of two or more gene sets sharing the same core group of genes, thus explaining the enrichment of each of the gene sets. Enrichment significance (*P*-value) is conveyed as node color intensity where red stands for upregulation in the CA piglets fed the MCT or the CTRL diets, and blue for downregulationas compared with the SA piglets. The node size represents the number of genes in the gene set

A second gene enrichment study was generated to demonstrate the effect of early association with microbiota of different complexities regarding the enrichment of gene sets expressed in the porcine oxyntic mucosa fed each of the two diets. Figure 3a visualizes the nodes of the gene sets of the Hallmark catalogue, enriched (color red) or impoverished (color blue) in samples from the CA piglets as compared to the SA piglets, and fed the MCT or the CTRL diet (represented by the center and the ring of each node, respectively). As compared to the SA piglets, the CA enriched genes in the sets related to both the interferon α and γ response when the CTRL diet was given while the same sets were impoverished by the CA with the MCT diet. The CA downregulated 5 gene sets in both the diets, all related to controlling the cell cycling, cellular transformations and the related stress-induced homeostatic mechanisms of DNA control and replication. Another 3 gene sets were also downregulated by the CA, including mTORC-related protein synthesis and cellular stress response related to the endoplasmic reticulum. Conversely, 3 gene sets were downgraded by the CA only when the CTRL diet was used, including the set related to protein secretion. The set of genes related to inflammatory response was enriched in the CA piglets fed only the CTRL diet while the TNFα and reactive oxygen species pathway sets were enriched in the SA piglets.

Figure 3b shows the nodes of the gene sets of the C5 catalogue (based on Gene Ontology), differentially enriched in the CA or the SA piglets, supplemented with medium chain fatty acids (MCTs) or not (CTRL), following the same scheme as Fig. 2a. Compared to the SA, the gene sets related to transmembrane receptor activity, including that of the neurotransmitters, were enriched in the CA piglets, particularly when fed the MCT diet (as demonstrated by the more intense red coloration). With the MCT diet, several gene sets related to anion channel activity were enriched by the CA as compared to the SA.

**Fig. 4:**
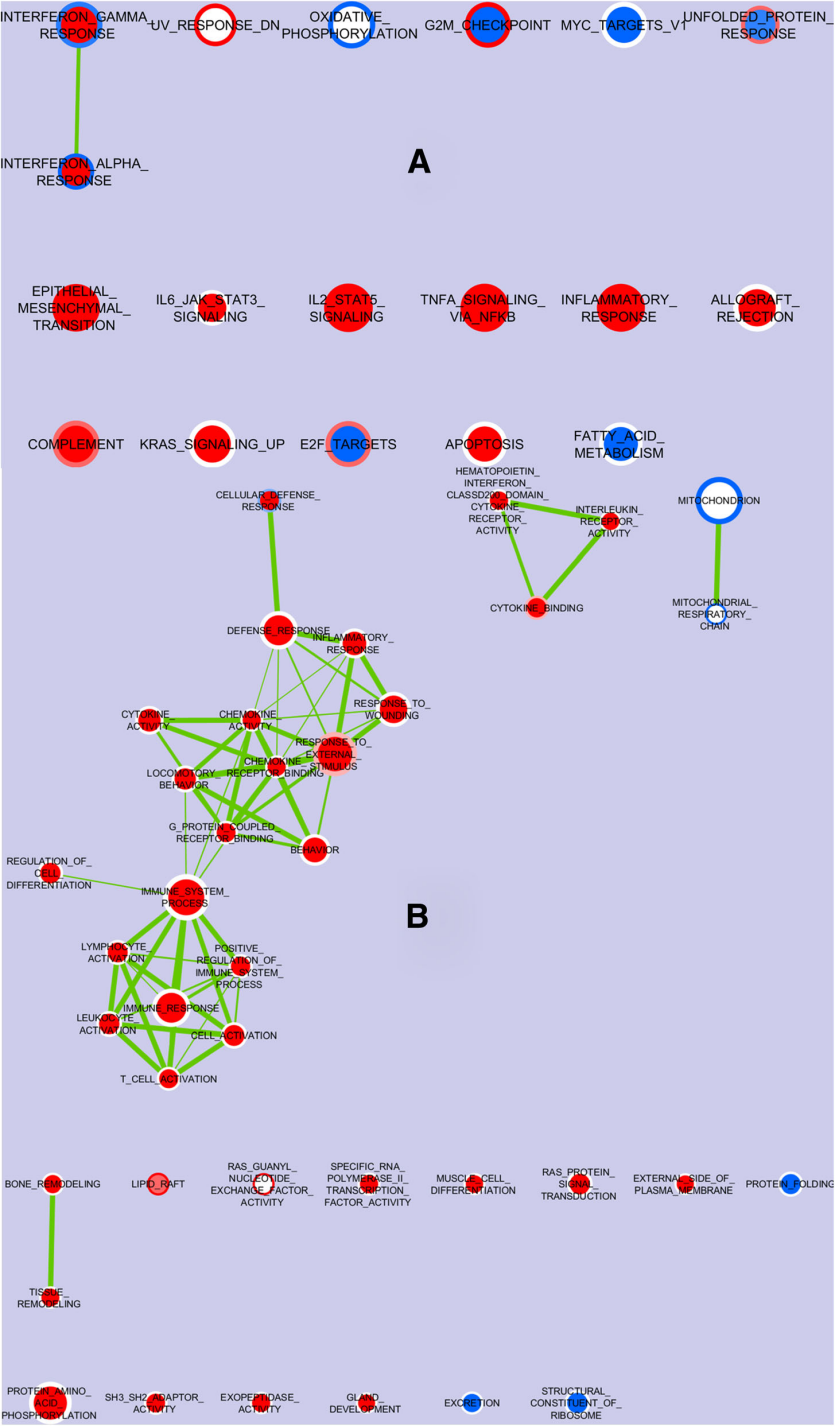
The effect of supplemention with a fortified diet with (MCT) or not (CTRL) on the nodes of the gene sets enriched in the oxyntic mucosa of piglets early associated with the CA) or the SA. **a** The entire picture for Hallmark gene set, summarizing the main biological states or processes; **b** Picture of nodes obtained with the more detailed GO gene set; the nodes represent the gene sets. The color on the ring represents the CA piglets while the color at the center of the node represents the SA piglets. The edges represent the link of two or more gene sets sharing the same core group of genes, thus explaining the enrichment of each of the gene sets. Enrichment significance (*P*-value) is conveyed as node color intensity where red stands for upregulation in the CA or the SA piglets fed the MCT diet, and blue for downregulation as compared with the CTRL-fed piglets. The node size represents the number of genes in the genen set

A third gene enrichment study was generated to demonstrate the effect of the different diets on the enrichment of the gene sets in the oxyntic mucosa from pigs which had an early association with microbiota of different complexities. Figure 4a shows the nodes of the gene sets of the Hallmark catalogue, enriched (color red) or impoverished (color blue) in samples from piglets fed the MCT diet compared to CTRL-fed piglets, and belonging to the CA or the SA group (represented by the ring and the center of each node, respectively). The MCT diet enriched gene sets of INFLAMMATORY_RESPONSE, TNFA_SIGNALING_VIA_NFKB, IL2_STAT5_SIGNALING, COMPLEMENT and also EPITHELIAL_MESENCHYMAL_TRANSITION, whatever the type of early association with the microbiota. As compared to the CTRL diet, feeding with the MCT diet impoverished genes in the sets related to both interferon α and γ response in the CA piglets while the same gene sets were enriched with the MCT diet in the SA piglets. Furthermore, ALLOGRAFT_REJECTION, IL6_JAK_STAT3_SIGNALING, APOPTOSIS, KRAS_SIGNALING_UP were also enriched by MCTs only in the SA piglets.

Several nodes were also observed with the more detailed GO catalogue (Fig. 4b) with the MCT diet in the SA piglets as compared to the CTRL diet: a large network of gene sets related to chemokine and cytokine activity and binding, immune cell activation and, in general, the positive regulation of the immune system process; sparse gene sets including those related to tissue modelling and differentiation.

## Discussion

The joint visualization of the results of the two experiments allowed demonstrating that some patterns of stimulation of gene groups are constantly related to the type of early association to microbiota, at least for the age range of our piglets. Conversely, other gene groups were regulated by the early treatment of pigs in a more age dependent manner. In general, early association with a complex starter microbiota tended to anticipate functional maturation while association with a simplified microbiota stimulated the cell cycles in the oxyntic mucosa.

### Proliferative response in the oxyntic mucosa

The local action of pathogenic bacteria can overactivate a range of growth signaling pathways within the gastric mucosa, thus altering the physiological balance between growth and apoptosis [17, 18]. On the contrary, in rats, the probiotic bacteria can have a restorative action on an acetic-induced gastric ulcer, reestablishing correct cell proliferation [19]. Bacteria can also produce putrescine and spermidine, the short-chain aliphatic amines involved in the regulation of cell proliferation and differentiation [20]. In our study, the early provision of a complex starter microbiota to the piglets favorably prevented the activation of pathways related to cycling activity and mitosis regulation steps (Fig. 1a) in the gastric mucosa at an age of two and three weeks as compared to the association with a simple microbiota. This may have been related to the observation that a short encounter with a complex microbiota in early life could be sufficient to influence the intestinal microbiota in the following weeks ([21] which was the same animal experimental design of trial 1. Nevertheless, the reduction of proliferative activity in the CA piglets was not accompanied by an insufficient maturation of the oxyntic mucosa but, on the contrary, several major genes implicated in the activity of the parietal cells or typical of the gastric mucosa were upregulated (Table 1).

### Acidic secretion function

Inside the stomach, the oxyntic mucosa is characterized by a very large and highly expressed array of genes for ion and water protein channels [16] as compared to the pyloric mucosa (and also to the intestinal mucosa). Thus, the observation that the new cured set of genes typical of the oxyntic mucosa was enriched in the CA piglets as compared to the SA piglets was important. The enrichment involved fewer genes in piglets sacrificed at an older age; especially at 3 wk of age no different expression was seen for the two genes, *ATP4A* and *ATP4B,* involved in the synthesis of H^+^/K^+^ −ATPase (Table 1). This may indicate that the potential of the proton pump to exchange luminal potassium with cytoplasmic hydronium was no different at this age between the CA and the SA piglets. Nevertheless, epidermal growth factor (EGF) is a growth factor important for gastric maturation and the constant upregulation of EGF may indicate that the local paracrine control was more active in the CA, including the stimulation of gastrin from the pyloric area [22]. Other potassium channels implicated in the K+ balance of parietal cells were also upregulated. In particular, several overlapping nodes of genes related to anion channel activity were upregulated in piglets fed MCTs which were early associated with a complex microbiota as compared to SA-associated piglets fed the same diet (Fig. 2b). This indirectly confirmed again that the oxyntic mucosa from the CA piglets was already more able to adapt to different dietary conditions. Notably, the most representative gene of these nodes for differential expression was the gene coding for chloride channel accessory 1 (*CLCA1*) which ranked 5^th^ in the list of upregulated genes. The CLCA1 and CLCA2 products in the stomach were characteristic of the luminal membranes of the murine gastric parietal cells [23] and also of surface mucosa.

### Maturation of gastric signaling, enteroendocrine control and neuronal controls

In the piglets that had been associated with a complex microbiota, particularly when fed the MCT diet, the connected nodes G_PROTEIN_COUPLED_RECEPTOR (GPCR)_ACTIVITY, RHODOPSIN_LIKE_RECEPTOR_ACTIVITY and NEUROTRANSMITTER_ _RECEPTOR_ACTIVITY were enriched of activated genes (Fig. 2b). Several GPCRs are important for the maturation of the gastric mucosa [24] and for the ghrelin-secreting cells [25]. In the list of upregulated genes of these nodes, the highest rank was for the gene for 5-hydroxytryptamine (Serotonin) receptor 1B, G protein-coupled (*HTR1B*). Serotonin is produced in the gastric mucosa by the enterochromaffin cells and the variation of this gene demonstrates the interacting role of early priming by the microbiota and the diet which controls the serotonergic signaling in the oxyntic mucosa. Some metabolites derived from the gut microbiota can modulate serotonin expression in the colon [26]. Furthermore, the gene for another serotonin receptor (number 4) was co-associated with the serotonin transporter gene across different states of microbial association colonization stages in the mouse colon [27].

The CA treatment enriched the complex of nodes related to neurotransmitter binding, to neurotransmitter receptor activity and to transmembrane receptor activity. The gastric parietal cells are innervated by secretomotor neurons and stimulated to release acid, and the same is also true for the chief cells which release pepsinogen. The gastric acid secretomotor neurons are cholinergic [28]. Thus, the CA could have better activated neuronal control of the gastric secretions, as indicated by its first rank in the list of the genes of the cholinergic receptor, nicotinic, alpha 2 (neuronal) (*CHRNA2*). Another high-ranking gene was the gamma-aminobutyric acid (GABA) A receptor, gamma 2 (*GABRG*2). In the stomach, the cholinergic enteric neurons present GABAergic neuron immunoreactivity; GABA may also be sensed as hormonal and paracrine signaling [29]. Interestingly, GABA is produced by several common gut commensals, including some lactic acid bacteria [30]. In fact, our observation could be associated with the presence of a more developed and complex gastric microbiota.

### Induction and regulation of the gastric immune system and local inflammation by both the microbiota and the diet

In general, it has been well demonstrated that, in mammals, the first week of life are extremely relevant for the induction of proper maturation of all the key actors of innate and acquired defenses in the gut, in functional dependence on the host antigenic experience. Specifically, in piglets, in the passage from the 2^nd^ to the 4^th^ wk of age, the intestinal mucosa becomes colonized by CD4 + T cells and the number of intraepithelial lymphocytes, and IgM + B cells start to appear in small numbers [31].

The degree of activation of different pathways related to the induction and the regulation of the gastric immune system and local inflammation by the microbiota changed with the age of the animals according to the type of previous association with microbiota and diet. With the control diet, the piglets sampled at three weeks of age which had been associated with a complex microbiota had overlapping enriched nodes related to interferon α and γ, cellular defense response and inflammatory response as compared to the SA piglets (Fig. 1a). This activation was also linked to more activity and the receptor binding activation of the cytokines (Fig. 1b). Of the genes in these groups, there was gene coding for chemokine (C-X-C motif) ligand 11 (*CXCL11*) which ranked 2^nd^ in the list of all genes. The chemotactic protein CXCL11 is involved in mature T-cell recruitment and is induced by interferon γ [32]. All the piglets were apparently healthy and, thus, this could be interpreted in the general situation of the higher functional maturity of the CA piglets. The presence of a remodeling phase in the older CA piglets versus the SA piglets could also be supported by the enrichment of gene sets COAGULATION and EPITHELIAL_MESENCHIMAL_ TRANSITION where the first ranking gene was matrix metalloproteinase 3 (*MMP3*) responsible for tissue remodeling [33]. The differential maturation of the immune cells associated with the gastric tissue of the CA piglets as compared to the SA piglets is also indicated by the higher expression of a gene not considered in the two GSEA catalogues, JCHAIN (Joining chain of multimeric IgA and IgM). The product of the JCHAIN is essential for the fusion of two monomer units of either IgM or IgA, also cooperating with their binding to the secretory component. This gene is induced by long-term bacterial colonization [34].

With MCTs, the interferon α and γ gene sets were stimulated in the SA piglets (Fig. 2a), but not in the CA piglets, indicating that the presence of MCTs in the diet could condition these piglets to a maturation such as that of the CTRL-fed CA piglets. These observations relating to the CTRL-fed piglets are, in some ways, in contrast with those obtained in the 1^st^ trial with the 2-week-old piglets. In this case, in addition to the already mentioned general mitotic stimulation, the sets of genes for cytokine/chemokine activity and chemokine receptor binding were enriched with the SA diet. Several chemokines characterized by the CXC motif were represented at the top of the core genes characterizing the enriched gene sets, including CXCL11. This led us to conclude that an early association of newborn piglets with a complex microbiota favorably prevents the activation of pathways related to cell division and inflammatory development in the gastric mucosa at an age of two weeks as compared to the association with a simple microbiota.

The diet containing an oil source of medium chain fatty acids in each group of piglets assigned to different early priming programs (3 wk of age, trial 2), in general, activated the inflammatory response, with the involvement of T cell cycling (indicated by the enrichment of IL2_STAT5_sSIGNALING together with TNFA_SIGNALING_VIA_NFKB) (Fig. 3a). This was also particularly associated with the activation of *CCL2* (Chemokine (C-C Motif) Ligand 2) and *CXCL10* (Chemokine (C-X-C Motif) Ligand 10) involved in monocyte chemotaxis and T-cell migration [35]. However, this was particularly evident when MCTs were given to the SA piglets, with a general enrichment of the nodes related to chemokine activity and cytokine binding (Fig. 3b). Interestingly, feeding piglets a diet supplemented with coconut oil increased the intestinal translocation of the bacterial enterotoxin as compared to the control diet or a polyunsaturated oil [36]. The Authors of this study explained this observation with the increased endocytosis of the enterotoxin favored by the innate immune receptor complex based on CD 14/Toll-like receptor 4 (TLR4)/ Lymphocyte Antigen 96. The Authors detected consistent expression of the *TLR4* gene in our data set and a similar action regarding the MCT diet cannot be excluded, with the consequent induction of the inflammatory response by the enterotoxin eventually present in the stomach and derived from resident or transient bacteria.

## Conclusions

The quality of early microbial colonization is important in promoting the functional maturation of the oxyntic mucosa in an age-dependent manner, as indicated by the variation of several genes typical of the oxyntic mucosa and of the gastric tissue in general. Furthermore, this was relevant for establishing a strong balance between the regulation of the immune system and the inflammatory response. The partial dietary substitution of unsaturated fatty acids with a medium chain fatty acid source may have affected the activation of several pathways addressed to the recruitment and the functional maturation of immune cells, especially when the piglets were early associated with a simplified starter microbiota. On the whole, these observations are important for the feed industry when designing strategies to stimulate the gut and immune system maturation of the piglet. Furthermore, the presence of an interaction of the complexity of early microbial colonization with the diet can explain the frequent variable response to early supplemented probiotics to the piglets and be relevant also as a general model for young human studies.

## Abbreviations

ATP4A: ATPase, H^+^/K^+^ exchanging, α polypeptide
ATP4B: ATPase, H^+^/K^+^ exchanging, α polypeptide
CA: Treatment with association with complex microbiota inoculum
CCL2: Chemokine (C-C Motif) Ligand 2
CHRNA2: Cholinergic receptor, nicotinic, alpha 2 (neuronal)
CLCA1: For chloride channel accessory 1
CTRL: Control diet
CXCL10: Chemokine (C-X-C Motif) Ligand 10
CXCL10: Chemokine (C-X-C Motif) Ligand 10
EGF: Epidermal growth factor
FDR: False discovery rate
GABRG2: Gamma-aminobutyric acid (GABA) A receptor, gamma 2
GO: Gene Ontology
HTRIB: for 5-hydroxytryptamine (Serotonin) receptor 1B, G protein-coupled
JCHAIN: Joining chain of multimeric IgA and IgM
KCNE2: Potassium voltage-gated channel, Isk-related family, member 2
KCNJ15: Potassium inwardly rectifying channel, subfamily J, member 15
LPL: Lipoprotein lipase
MCT: Diet with medium chain tryglicerides
MMP3: Matrix metalloproteinase 3 matrix metalloproteinase 3
NES: Normalized enrichment score
SA: Treatment with simplified microbiota
SLC2A4: Solute carrier family 2 (facilitated glucose transporter), member 4
SLC4A2: Anion exchanger 2
T1-T2: Trial 1 and Trial 2
TGFB: Transforming growth factor β
TLR4: Toll like receptor 4
